# Impact of comorbid asthma on severity of coronavirus disease (COVID-19)

**DOI:** 10.1038/s41598-020-77791-8

**Published:** 2020-12-11

**Authors:** Sang Chul Lee, Kang Ju Son, Chang Hoon Han, Ji Ye Jung, Seon Cheol Park

**Affiliations:** 1 Division of Pulmonology, Department of Internal Medicine, National Health Insurance Service Ilsan Hospital, Goyang-si, Gyeonggi-do Republic of Korea; 2 Department of Research and Analysis, National Health Insurance Service Ilsan Hospital, Goyang-si, Gyeonggi-do Republic of Korea; 3 Department of Biostatistics and Computing, Yonsei University Graduate School, Seoul, Republic of Korea; 4 Division of Pulmonary and Critical Care Medicine, Department of Internal Medicine, Severance Hospital, Yonsei University College of Medicine, Seoul, Republic of Korea

**Keywords:** Medical research, Risk factors

## Abstract

The severity of the coronavirus disease (COVID-19) is associated with various comorbidities. However, no studies have yet demonstrated the potential risk of respiratory failure and mortality in COVID-19 patients with pre-existing asthma. We selected 7272 adult COVID-19 patients from the Korean Health Insurance Review and Assessment COVID-19 database for this nationwide retrospective cohort study. Among these, 686 patients with asthma were assessed by their severities and evaluated by the clinical outcome of COVID-19 compared to patients without asthma. Of 7272 adult COVID-19 patients, 686 with asthma and 6586 without asthma were compared. Asthma was not a significant risk factor for respiratory failure or mortality among all COVID-19 patients (odds ratio [OR] = 0.99, *P* = 0.997 and OR = 1.06, *P* = 0.759) after adjusting for age, sex, and the Charlson comorbidity score. However, a history of acute exacerbation (OR = 2.63, *P* = 0.043) was significant risk factors for death among COVID-19 patients with asthma. Asthma is not a risk factor for poor prognosis of COVID-19. However, asthma patients who had any experience of acute exacerbation in the previous year before COVID-19 showed higher COVID-19-related mortality, especially in case of old age and male sex.

## Introduction

Severe acute respiratory syndrome coronavirus 2 (SARS-CoV-2) was identified as the cause of the coronavirus disease (COVID-19) outbreak in Wuhan, China, on January 9, 2020. Since the World Health Organization declared COVID-19 a pandemic, the number of cases has increased at an exponential rate. To date, there have been 19,369,210 confirmed cases around the world with 732,499 deaths^1^.

This novel single-stranded enveloped ribonucleic acid virus is the seventh known human coronavirus. It belongs to the same family that causes severe acute respiratory syndrome (SARS) and Middle East respiratory syndrome (MERS), which are transmitted through similar zoonoses. Although the virologic characteristics of SARS-CoV-2 are similar to those of MERS and SARS, it is highly contagious compared to them^2,3^. Symptoms of COVID-19 range from mild upper respiratory infections to severe pneumonia and death because of acute respiratory failure. To date, various host factors, such as old age, smoking, and comorbidities, are relevant to the prognosis of COVID-19^4–6^. In particular, the prognosis of patients with chronic obstructive pulmonary disease (COPD) is poor, and a promising mechanism by increased gene expression of angiotensin-converting enzyme 2 (ACE2) in the small airway epithelium has been explained^7^.

In addition to COPD, asthma is a major chronic airway disease, and its prevalence has been increasing worldwide since 1950^8,9^. More than 300 million people worldwide, including 25 million Americans, are affected by asthma. Asthma is a heterogeneous clinical syndrome that includes bronchial hyperresponsiveness, airway inflammation, and variable expiratory airflow limitation^10^. Generally, acute exacerbations of asthma following pneumonia can increase emergency room visits and admission rates, which could lead to poor outcomes, such as respiratory failure or death. Moreover, patients with severe asthma are exposed to prolonged, high-dose systemic corticosteroids, which could increase the mortality by developing systemic diseases, such as hypertension, diabetes, osteoporosis, and impaired humoral immunity^11,12^.

Since the emergence of COVID-19, scientists worldwide have been trying to understand the clinical, diagnostic, and prognostic aspects of the disease. However, no studies have been conducted on the prognostic outcomes in COVID-19 patients with pre-existing asthma. Therefore, this study analyzed the impact of asthma on the morbidities and mortalities of COVID-19 patients.

## Materials and methods

### Data source

In this study, we evaluated nationwide data regarding the clinical outcomes of COVID-19 in Korea between January 20 (diagnosis date of the first confirmed case) and May 27, 2020 using the administrative claims database of the Health Insurance Review and Assessment (HIRA) in South Korea. Since Korea has a national health insurance service, which provides universal coverage for almost every Korean citizens, HIRA has fully adjudicated medical and pharmacy claims for COVID-19 patients. The database of HIRA contains general demographic data, the 10th revision of the International Statistical Classification of Diseases and Related Health Problems (ICD-10), medications prescribed, and medical cost^13^.

### Study design

**Figure 1:**
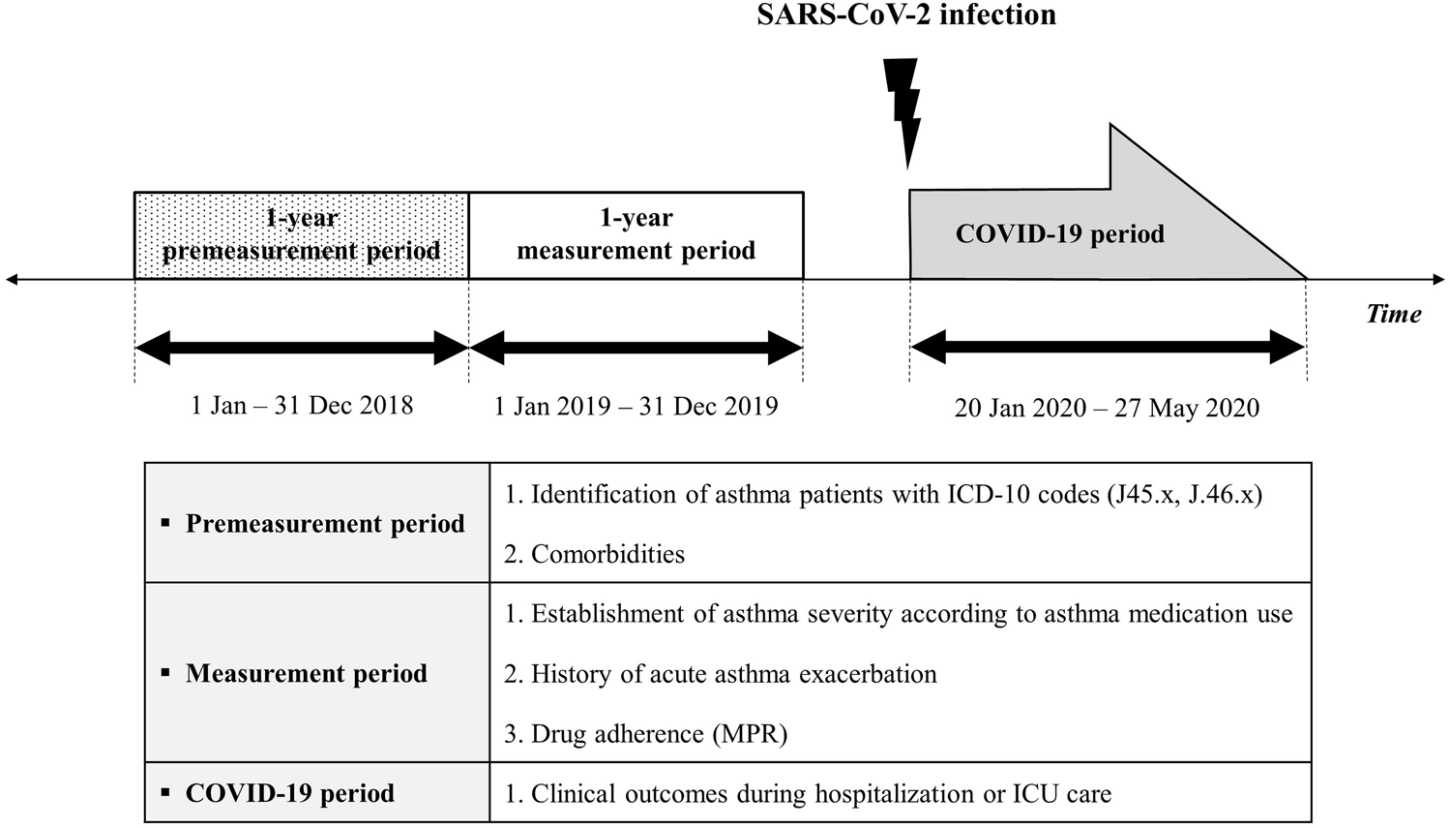
Study scheme. Information on the study population was collected from three different periods: the premeasurement period, measurement period, and coronavirus disease (COVID-19) period. The inclusion and exclusion criteria were adapted, and baseline characteristics of patients were assessed during the premeasurement period. Asthma severity, number of acute exacerbation(s), and medication possession ratio were evaluated during the measurement period. COVID-related morbidities and mortalities were evaluated during the COVID-19 period. COVID-19, coronavirus disease; SARS-CoV-2, severe acute respiratory syndrome coronavirus 2; ICD-10, 10th revision of the International Statistical Classification of Diseases and Related Health Problems; MPR, medication possession ratio; ICU, intensive care unit.

We conducted a retrospective cohort study in South Korea to assess the risk factors of respiratory failure or mortality in COVID-19 patients with asthma. The patients with COVID-19 were defined by the following diagnostic codes using the HIRA dataset: B342, B972, B18, U181, and U071. All diagnoses were confirmed by reverse transcription-polymerase chain reaction testing for SARS-CoV-2. From the initial screening to the evaluation of the clinical outcomes of COVID-19 patients, the study period was divided into the following three different periods: 1. the premeasurement period, 2. measurement period, 3. the COVID-19 period (Fig. 1). During the premeasurement period (January 1–December 31, 2018), the inclusion and exclusion criteria were adapted among the adult COVID-19 patients. Variables associated with patient characteristics were also assessed during this period. Asthma severity, number of acute exacerbations, and drug adherence were measured 1 year after the premeasurement period (measurement period: January 1–December 31, 2019). After confirmed cases of COVID-19 were identified, morbidities and mortalities were evaluated. To better identify the impact of asthma, other respiratory ailments, such as COPD, bronchiectasis, and interstitial lung disease, were not included in this study.

### Case identification

Within the HIRA database, 7590 confirmed cases of COVID-19 were initially screened. Among them, 7272 confirmed COVID-19 patients aged ≥ 20 years were finally included, and they were divided into the asthma and non-asthma groups. Asthma patients were identified based on diagnostic and treatment evidence. All asthma cases were included when both diagnostic codes (primary or secondary diagnostic code for asthma: J45.x, J46.x) and medication codes were simultaneously identified more than twice during the premeasurement period. Medication codes for inhaled corticosteroids (ICSs), inhaled long-acting β2-agonists (LABA inhalers), inhaled long-acting muscarinic antagonists (LAMA inhalers), fixed-dose combinations, such as ICS/LABA inhalers, oral β2-agonists, leukotriene antagonists, mast cell stabilizers, xanthine derivatives, and systemic corticosteroids, were used to evaluate medication use for asthma. Cases in which patients were under 20 years of age or those with an observation period of less than 1 year were excluded. Subsequently, 686 COVID-19 patients with asthma were compared with 6586 COVID-19 patients without asthma.

### Charlson comorbidity index

The Charlson comorbidity index (CCI) is a widely used prognostic model that predicts 1-year mortality risk depending on the individual severity of comorbidities. The necessary information to calculate the CCI was obtained through the diagnostic codes obtained during the premeasurement period. Then, the CCI was calculated by the summation of the scores of each comorbidity, as shown in Supplementary Table 1. We adapted and analyzed the CCI as a variable because it was suitable for measuring the impact of comorbidities on mortality using the health care administrative database, including ICD-10 codes^14,15^.

**Table 1:** Patient characteristics.

Characteristics	Non-asthma (n = 6586)	Asthma (n = 686)	*P*-value*	Asthma Severity		*P*-value^†^
				Mild (n = 614)	Moderate to severe (n = 72)	
**Age (years)**			< 0.001			0.002
20–39	2488 (37.7)	173 (25.3)		164 (26.7)	10 (13.8)	
40–59	2267 (34.4)	211 (30.7)		194 (31.6)	17 (23.6)	
≥ 60	1831 (27.8)	301 (43.8)		256 (41.6)	45 (62.5)	
**Sex, male**	2680 (40.6)	247 (36.0)	0.017	215 (35.0)	32 (44.4)	0.114
**Comorbidities**						
Chronic rhinitis	2951 (44.8)	599 (87.3)	< 0.001	541 (88.1)	58 (80.5)	0.068
Hypertension	1193 (18.1)	208 (30.3)	< 0.001	183 (29.8)	25 (34.7)	0.390
Diabetes	863 (13.1)	178 (25.9)	< 0.001	155 (25.2)	23 (31.9)	0.219
Dyslipidemia	1640 (24.9)	272 (39.6)	< 0.001	233 (37.9)	39 (54.1)	0.007
IHD	248 (3.7)	66 (9.6)	< 0.001	57 (9.2)	9 (12.5)	0.381
Heart failure	110 (1.6)	44 (6.4)	< 0.001	39 (6.3)	5 (6.9)	0.846
Malignancies	239 (3.6)	44 (6.4)	< 0.001	36 (5.8)	8 (11.1)	0.085
**CCI score**			< 0.001			0.027
0	3828 (58.1)	0 (0.0)		0 (0.0)	0 (0.0)	
1	1264 (19.1)	270 (39.3)		252 (41.0)	18 (25.0)	
2	653 (9.9)	147 (21.4)		129 (21.0)	18 (25.0)	
3	339 (5.1)	101 (14.7)		91 (14.8)	10 (13.8)	
≥ 4	502 (7.6)	168 (24.4)		142 (23.1)	26 (36.1)	
**MPR group**			–			< 0.001
Low	–	508 (81.6)		525 (85.5)	35 (48.6)	
Medium	–	30 (4.3)		23 (3.7)	7 (9.7)	
High	–	96 (13.9)		66 (10.7)	30 (41.6)	
**Number of acute exacerbations**			–			< 0.001
0	–	650 (94.7)		594 (96.7)	56 (77.7)	
1	–	19 (2.7)		11 (1.7)	8 (11.1)	
2	–	17 (2.4)		9 (1.4)	8 (11.1)	

Data are presented as numbers (%) or means ± standard deviations.

IHD, ischemic heart disease; CCI, Charlson comorbidity index; MPR, medication possession ratio.

*P-value for comparison between asthma and non-asthma groups.

^**†**^*P*-value for comparison between mild and moderate-severe groups.

### Assessment of asthma severity

During the measurement period, asthma severity was assessed based on the patient’s medication use according to the guidelines from the Global Initiatives for Asthma (GINA)^10^. Although the stepwise approach with dose modification of ICS or oral asthma medication(s) is the backbone of asthma treatment, there is a limitation in clearly distinguishing the precise dosage of ICS or ICS/LABA in the administrative claims database of HIRA. Hence, previous studies suggested a new definition of asthma severity to analyze the claims data^16,17^. According to the prescribed medication(s) during the premeasurement period, asthma severity was classified into three exclusive categories: mild, moderate, and severe. Asthma severity was defined as “mild” if the patient was prescribed at least one asthma medication, excluding ICS/LABA inhalers, low-dose systemic corticosteroids (defined as a prednisolone equivalent of < 10 mg/day for at least 2 weeks), and tiotropium. Asthma severity was defined as “moderate” if a patient was prescribed a low-dose or high-dose ICS/LABA inhaler, but not tiotropium or low-dose oral corticosteroids (OCSs). Asthma severity was defined as “severe” if a patient was prescribed an ICS/LABA inhaler and received at least one prescription of tiotropium or a low-dose OCS.

### Definition of acute exacerbation and medication possession ratio

Acute exacerbation was defined as the presence of primary or secondary diagnostic code for asthma (J45.x, J46.x), along with any of the following: (1) high-dose systemic corticosteroid use (≥ 30 mg, over 3 days consecutively), (2) hospitalization, or (3) emergency room visit. To measure the drug adherence for asthma medications, medication possession ratio (MPR) was used^18,19^. The MPR was calculated as the sum of the day’s supply for all medications, and divided by the time from first fill until the end of the measurement period^20^. Subsequently, patients were categorized into three adherence groups as follows: low (MPR < 50%), medium (MPR 50% to 80%), and high (MPR > 80%)^21^. Asthma medications, such as ICSs, LABA or LAMA inhalers, ICS/LABA inhalers, oral β2-agonists, leukotriene antagonists, mast cell stabilizers, xanthine derivatives, and systemic corticosteroids, were used to measure the MPR status.

### Clinical outcome and severity of COVID-19

The primary outcome was respiratory failure and mortality among COVID-19 patients. Cases of mechanical ventilation or extracorporeal membrane oxygenation (ECMO) were defined as patients who experienced respiratory failure. Other clinical outcomes, such as length of hospital stay, overall medical expenses, number of intensive care unit (ICU) care cases, and length of ICU care, were also evaluated. Depending on the clinical outcomes, study subjects were divided into two groups: severe and non-severe groups. Patients who had history of ICU care, mechanical ventilation, ECMO, or death, were classified as severe group. All other COVID-19 patients were classified as non-severe group.

### Statistical analyses

The baseline characteristics of each group were compared using the paired t-test and chi-square analysis. Subsequently, multivariate logistic regression analysis was performed to evaluate the association between the risk factors and COVID-19-related outcomes and was reported as adjusted odds ratio (OR) with 95% confidence interval (CI). Two models were used to assess the risk of respiratory failure and death, one analyzing the asthma group and the other analyzing moderate to severe asthma group as risk factors. All data transformations and statistical analyses were conducted using the Statistical Analysis System software V9.4 for Windows (SAS Institute, Cary, NC, USA). And all tests were two-sided, with a significance level of 0.05.

### Ethics approval

This study was approved by the Institutional Review Board of the National Health Insurance Service Ilsan Hospital and adhered to the tenets of the Declaration of Helsinki (NHIMC 2020-04-010). The requirement for informed consent was waived by Institutional Review Board of the National Health Insurance Service Ilsan Hospital because of the retrospective nature of this study. All personal identifying information of the study individuals was anonymous.

### Ethical declarations

This article does not contain any studies with human or animal subjects performed by any of the authors.

## Results

### Patient characteristics

**Figure 2:**
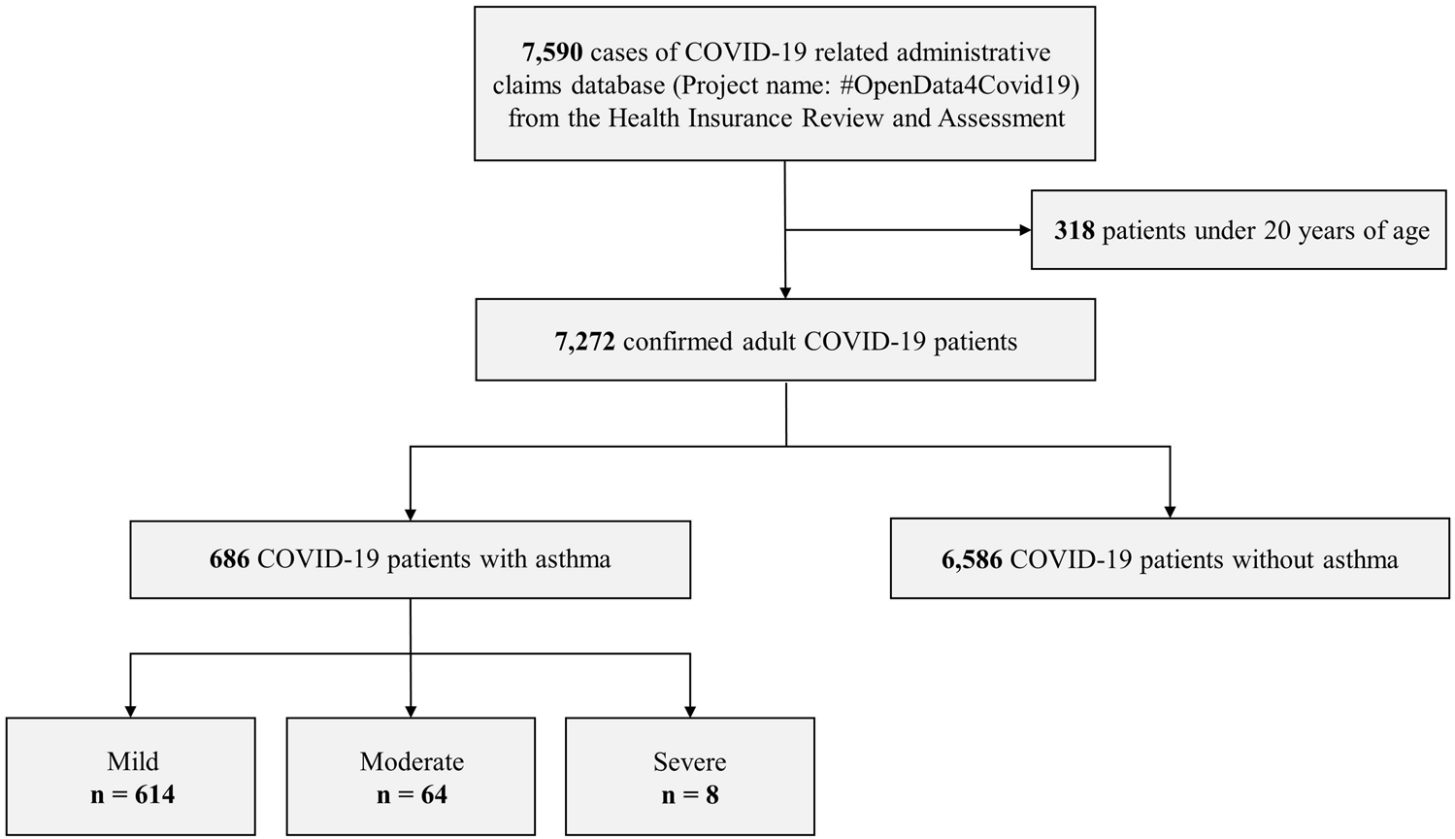
Flowchart of the study population. A total of 7590 coronavirus disease (COVID-19) patients were identified using the 10th revision of the International Statistical Classification of Diseases and Related Health Problems (ICD-10) diagnosis code. Among 7272 adult patients ≥ 20 years of age, 686 patients with asthma were identified using the ICD-10 code and medication use for asthma. Severity of asthma was classified according to the type of medication prescribed. COVID-19, coronavirus disease.

Of the total 7272 COVID-19 patients, 686 (9.4%) patients with asthma and 6586 (90.6%) without asthma were compared. Among the patients with asthma, 614 (89.5%), 64 (9.3%), and 8 (1.1%) were classified as “mild,” “moderate,” and “severe,” respectively (Fig. 2). Baseline demographics of the study population are shown in Table 1. The asthma group predominantly consisted of elderly patients over 60 years of age (43.8%), and a large number of young patients under 40 years of age (37.7%) was included in the non-asthma group. In both the groups, the number of more women was more than men (64.0% in asthma and 59.4% in non-asthma groups). In the asthma group, all comorbidities were observed more frequently, and the CCI score. When asthma severity was compared, the moderate to severe group showed a higher proportion of older patients and higher CCI score than the mild group. Additionally, the moderate to severe group showed a higher proportion of patients with medium to high MPR and more frequent history of acute exacerbation than the mild group.

### COVID-19-related morbidities and mortalities

COVID-19-related medical costs were higher in the asthma group than in the non-asthma group (5488.4 United states dollar [USD] vs. 4410.1 USD, *P* < 0.001) (Table 2). Although there was no difference in the hospitalization rates between the two groups, the length of hospital stay was longer in the asthma group (24.0 days vs. 22.3 days, *P* = 0.01). According to the severity classification, proportion of severe COVID-19 cases who showed severe clinical outcomes including ICU care, application of mechanical ventilation or ECMO, and death were higher in asthma group (9.1% vs. 4.6%, *P* < 0.001). In asthma patients, there was no significant difference of COVID-19 severity depending on asthma severity. In detail, ICU care rates in the asthma group were significantly higher (3.9% vs. 2.4%, *P* = 0.022) than in the non-asthma group. Among them, mechanical ventilation or ECMO, which indicated respiratory failure, was also frequent in the asthma group. In addition, the overall mortality rate was significantly higher in the asthma group (6.4% vs. 2.7%, *P* < 0.001). Among the asthma severity groups, patients with moderate to severe asthma had higher medical expenses than the mild asthma group during COVID-19 treatment (6577.4 USD vs. 5360.6 USD, *P* < 0.001). The proportion of deaths was also significantly higher in the moderate to severe asthma group than in the mild asthma group (13.8% vs. 5.5%, *P* = 0.006).

**Table 2:** Clinical outcomes of coronavirus disease between asthma and non-asthma groups.

Variable	Non-asthma (n = 6586)	Asthma (n = 686)	*P*-value*	Asthma severity		*P*-value^†^
				Mild (n = 614)	Moderate to severe (n = 72)	
**COVID-19-related medical cost (USD)**	4410.1 ± 5560.4	5488.4 ± 8412.9	< 0.001	5360.6 ± 7062.1	6577.4 ± 16,720.7	< 0.001
**Admission**	6169 (93.6)	642 (93.5)	0.932	575 (93.6)	67 (93.0)	0.846
Length of stay	22.3 ± 13.4	24.0 ± 15.4	0.010	23.7 ± 15.3	25.8 ± 15.8	0.312
**COVID-19 severity**			< 0.001			0.143
Non-severe	6278 (95.3)	623 (90.8)		561 (91.3)	62 (86.1)	
Severe	308 (4.6)	63 (9.1)		53 (8.6)	10 (13.8)	
**ICU care**	163 (2.4)	27 (3.9)	0.022	25 (4.0)	2 (2.7)	0.593
Length of stay	10.7 ± 9.1	9.8 ± 7.6	0.626	10.1 ± 7.8	6.5 ± 5.9	0.531
Application of mechanical ventilation	99 (1.5)	19 (2.7)	0.012	17 (2.7)	2 (2.7)	0.996
Application of ECMO	15 (0.2)	6 (0.8)	0.002	5 (0.8)	1 (1.3)	0.620
**Death**	183 (2.7)	44 (6.4)	< 0.001	34 (5.5)	10 (13.8)	0.006

Data are presented as numbers (%) or means ± standard deviations.

*P-value for comparison between asthma and non-asthma groups.

^**†**^*P*-value for comparison between mild and moderate-severe groups.

COVID-19, coronavirus disease; USD, United states dollar; ICU, intensive care unit; ECMO, extracorporeal membrane oxygenation.

### Risk factors for respiratory failure and mortality in total COVID-19 patients

Age (OR = 1.06, *P* < 0.001 and OR = 1.12, *P* < 0.001), male sex (OR = 2.31, *P* < 0.001 and OR = 2.46, *P* < 0.001), and the CCI score (OR = 1.13, *P* = 0.001 and OR = 1.18, *P* < 0.001) were identified as independent risk factors for respiratory failure and death in COVID-19 patients. Asthma was not a risk factor for respiratory failure or death, but moderate to severe asthma showed a non-significant high risk of death (OR = 1.98, *P* = 0.065) (Table 3). Multivariate analysis using separate comorbidities instead of CCI score showed that diabetes and heart failure were independent risk factors for mortality (OR = 2.02, *P* < 0.001 and OR = 1.98, *P* = 0.127; respectively) (Supplementary Table 2). Among the patients with asthma, age was a significant risk factor for both respiratory failure and death (OR = 1.05, *P* < 0.001 and OR = 1.10, *P* < 0.001, respectively). Additionally, male sex (OR = 2.26, *P* = 0.021) and a history of acute exacerbation of asthma within 1 year (OR = 2.63, *P* = 0.043) of COVID-19 infection showed increased mortality (Table 4).

**Table 3:** Multivariate analyses of risk factors associated respiratory failure and mortality among total study population.

Variable	Respiratory failure risk		Mortality	
	Adjusted OR (95% CI)	*P*-value	Adjusted OR (95% CI)	*P*-value
**Model 1**				
Age	1.06 (1.05–1.07)	< 0.001	1.12 (1.11–1.14)	< 0.001
Sex, male	2.31 (1.58–3.37)	< 0.001	2.46 (1.82–3.33)	< 0.001
CCI	1.13 (1.05–1.23)	0.001	1.18 (1.11–1.25)	< 0.001
Asthma	0.99 (0.58–1.70)	0.997	1.06 (0.71–1.59)	0.759
**Model 2**^**a**^				
Moderate-severe asthma	0.66 (0.15–2.82)	0.581	1.71 (0.78–3.73)	0.173

OR, odds ratio; CI, confidence interval; CCI, Charlson comorbidity score.

^a^Adjusted for age, sex (male), and CCI.

**Table 4:** Multivariate analyses of risk factors associated mortality in patients with asthma.

Variable	Respiratory failure risk		Mortality	
	Adjusted OR (95% CI)	*P*-value	Adjusted OR (95% CI)	*P*-value
Age	1.05 (1.02–1.08)	< 0.001	1.10 (1.07–1.14)	< 0.001
Sex, male	1.00 (0.38–2.62)	0.985	2.26 (1.12–4.54)	0.021
**Severity**				
Mild	1 (Reference)		1 (Reference)	
Moderate-severe	0.81 (0.17–3.76)	0.795	1.33 (0.54–3.30)	0.526
**Number of acute exacerbation(s)**				
0	1 (Reference)		1 (Reference)	
≥ 1	0.42 (0.05–3.58)	0.432	2.63 (1.02–6.72)	0.043

OR, odds ratio; CI, confidence interval; MPR, medication possession ratio.

## Discussion

Currently, there is a lack of studies on the prognostic outcomes in COVID-19 patients with pre-existing asthma. Recently, several studies have suggested possible non-harmful effects of asthma on the clinical outcomes of COVID-19. A retrospective study by Zhao et al. evaluated the risk factors among 548 COVID-19 inpatients in Wuhan and reported that there were few patients with asthma and the risk was consistent with severe COVID-19 cases^22^. A subsequent epidemiological study and literature review also supported that the comorbidity rates of asthma were significantly lower than the reported prevalence of asthma in the respective regions^23^. However, the proportion of asthma patients among the total COVID-19 patients in our study was 9.4%, which is relatively higher than the previously known prevalence in Asian-Pacific countries^24,25^. This prevalence was similar to other studies in the USA or UK, which also showed high comorbidity rates of asthma (12.5% and 14%, respectively)^26,27^. Previously, Park et al. demonstrated that elderly and female patients showed a higher prevalence of asthma^28^. Hence, the comorbidity rate of asthma could be variable according to the composition of the study population. In South Korea, there was a large cluster of COVID-19 infection within a certain religious group, which comprised of twice the number of women worshippers than men^29^. The number of transmissions linked to this religious facility was over half of the total COVID-19 patients (4363; 59.9%) in South Korea. However, unfortunately, detailed information about transmission during this religious meeting was not well known because of the secret property. In addition, susceptibility to infectious diseases could be considered. Men are generally more susceptible to infection with diverse pathogens than women^30,31^. Though, there is also evidence that women are more susceptible to infectious diseases, which could possibly explain why the number of women patients in this study was higher than that of men^32,33^. Therefore, a large number of women were included in our study, which could have increased the proportion of asthma patients.

Compared to the non-asthma group, asthma group showed a larger amount of COVID-19-related medical expenses and higher proportion of respiratory failure, which led to the application of mechanical ventilation or ECMO. Because the median age and number of comorbidities were higher in the asthma group, the length of stay and frequencies of ICU admission were also high, which could contribute to the increased medical costs. However, multivariate analysis showed that asthma was not a significant risk factor for respiratory failure and death in COVID-19. Analysis after excluding the patients with mild asthma, even if there was an increasing tendency of OR in mortality, showed no statistical significance in patients with moderate to severe asthma. As we adapted an operative definition to distinguish asthma severity, relatively mild symptomatic patients could have been included in the moderate group. A small number of patients with moderate to severe asthma could also be the reason of this non-significance.

In addition, the role of ACE2 should be addressed to explain the relationship between asthma and COVID-19. The ACE2 receptor-binding domain is responsible for the first-stage host cell entry of the novel coronavirus^34,35^. Many studies have demonstrated the upregulation of the ACE2 receptor in current smokers with COPD^36,37^. Studies about the association between asthma and ACE2 expression are scarce; however, there are several studies on reduced ACE2 expression by type 2 inflammatory processes, which could lead to a decreased susceptibility to infection^22,23,38,39^. These studies suggested that the expression of ACE2 is likely to be regulated reciprocally by interferons and Type 2 cytokines, especially in allergic asthma. This could explain why asthma was not associated with poor outcomes, including respiratory failure and death.

In contrast, among the asthma patients, age, male sex, and a history of acute exacerbation were revealed as significant risk factors for mortality. Patients with a history of acute exacerbation of asthma during the previous year had a more than twofold higher risk of mortality than the patients without any exacerbation history. The current status of asthma control could impact future exacerbation and vice versa^40,41^. Moreover, acute exacerbations often lead to emergency room visits or hospitalization, which could increase the risk of COVID-19. Therefore, asthma patients with a recent exacerbation history should be considered as high-risk patients with poor prognostic outcome for COVID-19.

Although CCI was not an independent risk factor for poor prognostic outcomes, the diabetes significantly increased COVID-19 related mortality by twofolds of risk in consistent with other studies^42,43^. It is not yet known whether people with diabetes are more susceptible to COVID-19, but several studies have reported a greater risk of severe COVID-19 in diabetic patients. Dysfunctional and pro-inflammatory cytokine responses, such as elevation of IL-1, IL-6, and tumor necrosis factor-α, are thought to be the possible reason^44–46^.

Recently, two studies about asthma patients with COVID-19 in South Korea using HIRA database were published. Yang et al. analyzed entire cohort who underwent COVID-19 testing and reported that non-allergic asthma patients showed greater risk of susceptibility to COVID-19 infection and severe clinical outcomes of COVID-19, such as ICU admission, application of mechanical ventilation, or death^47^. Because they extracted asthma patients with ICD-10 codes only, study population could have included those without pure asthma comorbidity. In addition, Choi et al. also reported higher mortality in asthma patients than in non-asthma patients among COVID-19 patients^48^. However, asthma itself and its severity showed no significant relationship with poor outcome of COVID-19. Compared to these studies, we have adopted operative definition for stratification of unique asthma severity to overcome the limitation of medical claim database. Then, we demonstrated the clinical impact of recent exacerbation history of asthma on COVID-19 outcomes.

However, there were several limitations to this study. First, because of using administrative claims database, there is a potential for inaccuracies in coding and incompleteness of medical records. Moreover, information about smoking status, chest X-ray, and laboratory data were not available, so it was not possible to classify the severity of COVID-19 like previously reported guideline or studies^49–51^. Second, there were differences of baseline characteristics between asthmatics and non-asthmatics. Asthma group was older and showed higher CCI score, which could be the reason why asthma group showed significantly higher medical expenses and mortality although the multivariate analyses were conducted including age and CCI as covariates. Third, we used an operational definition to distinguish asthma severity, which may not fully reflect the current GINA steps. In our study, all patients who used an ICS/LABA inhaler were classified as moderate or higher regardless of the ICS dosage and frequency of inhaler use. Therefore, patients whose severity was GINA steps 2–4 could not be clearly distinguished. In addition, spirometry data, information on symptom variation (daytime or nocturnal symptoms), and optimal use of a controller or reliever inhaler, which are usually used to assess asthma control status, could not be measured in our study design. This may indicate that asthma severity with our operational definition could be underestimated compared to that of the GINA recommendation. Lastly, although systemic steroid use could be a risk factor for poor prognostic outcome, our study could not analyze it. The effect of systemic steroid use and dose-dependent relationship with prognostic outcome should be evaluated in future studies. Fourth, we could not assess the atopic status of individual asthma patients. To evaluate the protective effect of T helper 2 cell-mediated inflammation on the ACE expression of the airway cells in clinical practice, further studies, including laboratory results, which could distinguish among the phenotypes of asthma, are needed.

## Conclusions

Asthma itself is not a risk factor for poor prognosis of COVID-19. Elderly, males (only in asthma patients), or those who have multiple comorbidities need extra attention to avoid disease progression to respiratory failure or death. The mortality rate could be higher if the patients had experienced acute exacerbation in the previous year of COVID-19 infection, especially in case of old age and male sex. Therefore, special concerns for maintaining asthma control during the COVID-19 pandemic are needed to minimize the risk of acute exacerbation, which increases COVID-19-related mortality.

## Supplementary information

Supplementary Information.

## Abbreviations

COVID-19: Coronavirus disease
SARS-CoV-2: Severe acute respiratory syndrome coronavirus 2
SARS: Severe acute respiratory syndrome
MERS: Middle East respiratory syndrome
COPD: Chronic obstructive pulmonary disease
ACE2: Angiotensin-converting enzyme 2
HIRA: Health Insurance Review and Assessment
ICD-10: International Statistical Classification of Diseases and Related Health Problems
ICSs: Inhaled corticosteroids
LABA: Inhaled long-acting β2-agonists
LAMA: Long-acting muscarinic antagonists
CCI: Charlson comorbidity index
GINA: Global Initiatives for Asthma
OCSs: Low-dose oral corticosteroids
MPR: Medication possession ratio
