# Supplementary material for: Impact of comorbid asthma on severity of coronavirus disease (COVID-19)
Source: Sci Rep. 2020 Dec 11;10:21805. doi: 10.1038/s41598-020-77791-8 (PMC7733453; doi:10.1038/s41598-020-77791-8)
Supplement: Supplementary file 1 — Supplementary Information. [file 41598_2020_77791_MOESM1_ESM.pdf]

Impact of Comorbid Asthma on Severity of Coronavirus Disease (COVID-19)

Author names

Sang Chul Lee,[1*] Kang Ju Son,[2,3*] Chang Hoon Han,[1] Ji Ye Jung,[4†] Seon Cheol Park[1†]

Affiliations

[1]*Division of Pulmonology, Department of Internal Medicine, National Health Insurance Service Ilsan Hospital, Goyang-si, Gyeonggi-do, Republic of Korea*

[2]*Department of Research and Analysis, National Health Insurance Service Ilsan Hospital, Goyang-si, Gyeonggi-do, Republic of Korea*

[3]*Department of Biostatistics and Computing, Yonsei University Graduate School, Seoul, Republic of Korea*

[4]*Division of Pulmonary and Critical Care Medicine, Department of Internal Medicine, Severance Hospital, Yonsei University College of Medicine, Seoul, Republic of Korea*

* These authors contributed equally to this work as first authors.

[†] These authors contributed equally to this work as corresponding authors.

Corresponding author

Ji Ye Jung, MD, PhD

Division of Pulmonary and Critical Care Medicine, Department of Internal Medicine,

Severance Hospital, Yonsei University College of Medicine, Seoul 03722, Republic of Korea

Telephone number: 82-10-3141-2576; Fax number: 82-2-2228-2273

E-mail: stopyes@yuhs.ac

Seon Cheol Park, MD, PhD

Division of Pulmonology, Department of Internal Medicine,

National Health Insurance Service Ilsan Hospital, Goyang-si, Gyeonggi-do, Republic of Korea

Telephone number: 82-10-3793-1173; Fax number: 82-31-900-3663

E-mail: parksc@nhimc.or.kr

Supplementary Table 1. Determinants of Charlson comorbidity index

Score	Condition
1	Myocardial infarction
1	Congestive heart failure
1	Peripheral vascular disease
1	Cerebrovascular disease
1	Dementia
1	Chronic pulmonary disease
1	Connective tissue disease
1	Peptic ulcer disease
1	Mild liver disease
2	Hemiplegia
2	Moderate or severe renal disease
2	Diabetes with end-organ damage
2	Any malignancy without metastasis
2	Leukemia
2	Lymphoma
3	Moderate or severe liver disease
6	Metastatic solid malignancy
6	Acquired immune deficiency syndrome (AIDS)

Supplementary Table 2. Multivariate analyses of risk factors associated respiratory failure and mortality among total study population (analyzed with using separate comorbidities on behalf of CCI)

Variable	Respiratory failure risk		Mortality	
	**Adjusted OR (95% CI)**	***P*-value**	**Adjusted OR (95% CI)**	***P*-value**
Model 1				
Age	1.06 (1.04-1.07)	<0.001	1.12 (1.10-1.13)	<0.001
Sex, male	2.32 (1.59-3.39)	<0.001	2.51 (1.85-3.42)	<0.001
Hypertension	1.55 (0.99-2.43)	0.053	1.39 (0.99-1.96)	0.056
Diabetes	1.44 (0.93-2.24)	0.099	2.02 (1.44-2.84)	<0.001
Dyslipidemia	1.06 (0.68-1.66)	0.781	0.76 (0.53-1.08)	0.127
IHD	0.88 (0.47-1.63)	0.694	0.90 (0.57-1.44)	0.686
Heart failure	1.50 (0.74-3.05)	0.256	1.98 (1.17-3.33)	0.010
Malignancies	1.18 (0.60-2.29)	0.621	1.01 (0.59-1.72)	0.965
Asthma	1.04 (0.60-1.79)	0.877	1.28 (0.84-1.94)	0.240
Model 2*				
Moderate-severe asthma	0.72 (0.13-3.09)	0.672	2.12 (0.97-4.64))	0.059

*Adjusted for age, sex (male), hypertension, diabetes, dyslipidemia, IHD, heart failure, and mailgnancies
CCI, Charlson comorbidity index; OR, odds ratio; CI, confidence interval; IHD, ischemic heart disease
